# Thromboembolic risk with IVIg: Incidence and risk factors in patients with inflammatory neuropathy

**DOI:** 10.1212/WNL.0000000000008742

**Published:** 2020-02-11

**Authors:** Mahima Kapoor, Jennifer Spillane, Christina Englezou, Scherezade Sarri-Gonzalez, Robert Bell, Alexander Rossor, Hadi Manji, Mary M. Reilly, Michael P. Lunn, Aisling Carr

**Affiliations:** From the National Hospital of Neurology and Neurosurgery (J.S., C.E., S.S.-G., M.P.L., A.C.); MRC Centre for Neuromuscular Diseases, Department of Neuromuscular Diseases (M.K., A.R., H.M., M.M.R.), UCL Institute of Neurology; Department of Cardiology (R.B.), University College London Hospital; and Department of Neuroimmunology (M.P.L.), Institute of Neurology, London, UK.

## Abstract

Our objective was to evaluate whether IV immunoglobulin (IVIg) increases the risk of thromboembolic events in neurology outpatients with inflammatory neuropathies, as there is conflicting evidence supporting this hypothesis, mainly from non-neurologic cohorts. We investigated this question over 30 months in our cohort of patients with inflammatory neuropathies receiving regular IVIg and found a greater incidence of arterial and venous thromboembolic events than population-based rates determined by hospital admissions data. Vascular risk factors were more common in the event group but there were no IVIg administration factors that contributed to the risk. This study suggests that IVIg may have a small but contributory role in determining thromboembolic risk in the inflammatory neuropathy cohort and more evidence is required before it is clear whether the current primary prevention guidelines are appropriate in this group of patients.

## Introduction

Immune-mediated neuropathies are commonly managed with long-term IV immunoglobulin (IVIg). Adverse events associated with IVIg are usually mild and transient and include changes in blood pressure, tachycardia, mild flu-like symptoms, and headache. More serious adverse events are rare and include acute renal failure, aseptic meningitis, acute anaphylactic reactions, and hemolytic anaemia.^1^ An association between IVIg and either arterial or venous thromboembolic events (TEE) was suggested as long ago as 1986.^2^ Since then, studies looking at the incidence of IVIg-associated TEE have provided a wide range of rates from 3% to 11.2%.^3,4^

There are a number of pathogenic changes that occur following IVIg administration that could theoretically predispose to an increased TEE risk including an increase in serum viscosity, vasospasm, the release of vasoactive cytokines and clotting factors, sudden intravascular compartment expansion, and changes in venous compliance resulting in reduced capillary blood flow.^1^ However, the underlying characteristics of the individual and the disease being treated are likely to contribute and might explain some of the variation in observed rates.

A study looking specifically at inflammatory neuropathy patients receiving IVIg found that 11.3% of patients had a thromboembolic event over a 2-year period.^4^ Those who had an event were more likely to have a history of coronary artery disease, poor mobility, and cardiovascular risk factors than those who had no events. A dose of greater than 35 g/d was also associated with an increased risk of an event, although the total dose of IVIg per course was not. Clinically based studies tend to suggest an increased TEE risk but are limited by small numbers. The larger, prescription database studies are potentially biased by underreporting and are difficult to validate. Analysis of such data from the US health insurance records suggests a lower rate of thromboembolic events in otherwise high vascular risk individuals receiving IVIg and that IVIg may be protective due to its anti-inflammatory effects.^3^

Given this discrepancy, the risk of TEE events in patients on long-term IVIg remains uncertain and there are no guidelines about how the risk can be mitigated. In 2013, the Food and Drug Administration issued a black box warning for thrombosis related to human immunoglobulin products.^5^ They also suggest care should be taken in patients deemed to be at high risk of TEE to ensure adequate hydration and suggest monitoring baseline viscosity in patients at risk of hyperviscosity. There are inadequate epidemiologic and scientific data to support this advice.

## Aim

The National Hospital for Neurology and Neurosurgery (NHNN) has a large cohort of well-characterized and closely monitored patients with inflammatory neuropathies (predominantly chronic inflammatory demyelinating polyneuropathy [CIDP] and multifocal motor neuropathy [MMN] with conduction block) managed with regular, long-term IVIg. Our aim was to ascertain the frequency of TEE in our patients and to investigate patient and treatment factors that potentially increase risk.

## Methods

All patients with inflammatory neuropathy on regular IVIg between January 1, 2014, and July 31, 2016, were identified from our IVIg database and a retrospective chart review was performed. Patients treated acutely for Guillain-Barré syndrome (acute inflammatory demyelinating polyneuropathy) were excluded. Diagnosis, IVIg dose, frequency, vascular risk factors, pre- and post-treatment IgG levels, and plasma viscosity were recorded. Patients who had a TEE were identified from case note review and direct communication with the clinicians involved. Population-based TEE rates were obtained from contemporaneous hospital admissions data provided by National Health Service (NHS) Digital. NHS Digital is responsible for collecting and publishing data from the health and social care system in England and the hospital admission data are derived from the Hospital Episodes Statistics data warehouse where all admissions, appointments, and attendances for patients at NHS hospitals in England are recorded.^6,7^

QRISK2 is an assessment tool used in primary care to assess the 10-year risk of developing cardiovascular disease (CVD). A CVD risk score of 10% or more is a cutoff for considering statin treatment for primary prevention of CVD.^8^

### Standard protocol approvals, registrations, and patient consents

This study was approved by the University College London Hospital Trust (UCLH) clinical governance ethics and consent framework.

Patient consent was waived for this retrospective audit.

### Data availability

The neurology cohort data that support the findings of this study are available from the corresponding author upon reasonable request. Hospital admissions data on NHS Digital are freely available at digital.nhs.uk.

## Results

A total of 112 patients were included. CIDP (61 patients) and MMN with conduction block (41) were the commonest indications; others included chronic immune sensory polyradiculopathy,^6^ dorsal root ganglionopathy,^2^ CANOMAD (chronic ataxic neuropathy ophthalmoplegia M-protein agglutination disialosyl antibodies),^1^ and small fiber neuropathy.^1^ Patients received a mean (SD) dose of 1.6 (1.2) g/kg/mo at a mean interval of 4.4 (3.0), range 1–18 weeks.

### Thromboembolic events

Twelve TEEs were documented during the study period: 6 myocardial infarcts (MI), 2 strokes, and individual occurrences of deep vein thrombosis, pulmonary embolus, and superior vena cava obstruction secondary to thrombosis. None of the events occurred during or after the first course of IVIg. The incidence rates for any TEE, and arterial events or venous events considered separately, were markedly higher in our IVIg cohort compared to the general population (table). In the cohort, 85 (76%) patients mobilized independently, 13 (12%) used a single-point stick, 4 (3%) used 2 sticks, and 7 (6%) used a wheelchair; in the 11 patients with TEEs, 7 (64%) mobilized independently, 2 (18%) used 2 sticks, 2 (18%) used a wheelchair, and mobility status was unknown in 2 (18%) patients at time of event.

**Table:**
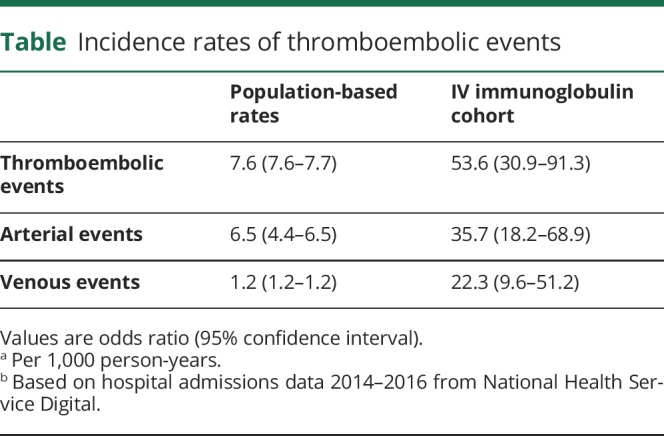
Incidence rates of thromboembolic events

### Cardiovascular risk factors

Vascular risk factors were more common in the event group, including age, hypertension, diabetes, and hypercholesterolemia (*p* < 0.05). Smoking status was not different between the 2 groups (*p* = 0.67); of note, smoking rates were low in both groups (>75% nonsmokers).

In our group, the QRISK2 score was higher in those who had events (*p* < 0.05) (figure). However, 1 individual had an event despite a score of less than 10%. This patient had commenced on strontium and hormone replacement therapy for bone protection after having a pathologic fracture during an unsuccessful trial of corticosteroids for her neuropathy; she had a right-sided lacunar infarct within 2 weeks of her second IVIg treatment. The hematology opinion was that the cumulative procoagulant effect of these 3 treatments was the cause of her stroke. Another patient had a complex, multifactorial cause for TEE. This patient had an MI secondary to an unusual left circumflex artery occlusion with otherwise patent coronary vasculature. Extensive cardiologic investigation concluded that this embolic event was related to the combination of the procoagulant effect of IVIg, an indwelling central venous access device to facilitate home IVIg treatment, and a patent foramen ovale identified on bubble contrast echocardiography.

**Figure:**
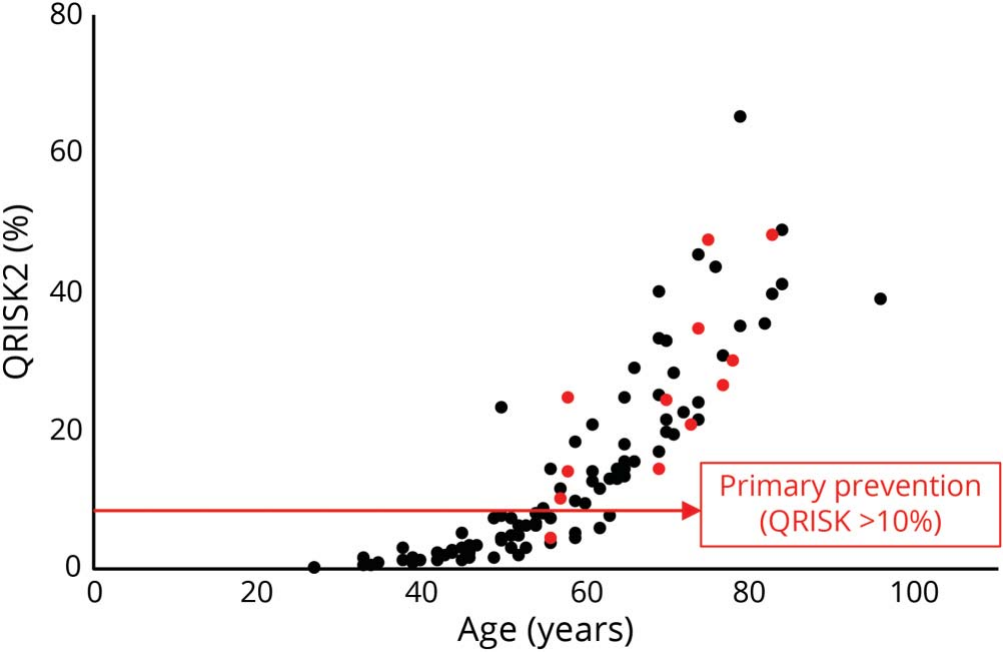
QRISK2 scores in those who did (red) and did not (black) have an event

### IVIg-related factors

There was no difference in the average monthly IVIg dose (*p* = 0.69) or daily dose in g/d (*p* = 0.28). There was a smaller treatment-related change in plasma IgG level (*p* < 0.05) and lower post-treatment plasma viscosity in those who had an event (*p* < 0.05).

## Discussion

We did not identify a relationship between TEE and factors directly related to IVIg administration but there was a higher rate of vascular risk factors in the event group. This suggests that IVIg confers a small but contributory thromboembolic risk in the inflammatory neuropathy cohort and that we should screen for vascular risk factors prior to IVIg commencement and treat them assertively, perhaps with a lower threshold for primary prevention than in the general population. In those without obvious, modifiable vascular risk factors, other procoagulant risks should be considered. Recording of suspected adverse events is essential for detecting unrecognized trends and hazards. Interestingly, the National Immunoglobulin Database, which records all clinical and administration data regarding the use of immunoglobulin by the NHS, only has 4 TEEs recorded as adverse events since its inception in 2010 (email correspondence).

These data support the concern that incidence of TEE is higher in patients receiving long-term IVIg compared to population-based estimates and that large database studies are likely to underestimate this risk due to underreporting.

## Glossary

CIDP: chronic inflammatory demyelinating polyneuropathy
CVD: cardiovascular disease
IVIg: IV immunoglobulin
MI: myocardial infarct
MMN: multifocal motor neuropathy
NHS: National Health Service
TEE: thromboembolic event

## References

[1] Kazatchkine MD, Kaveri SV. Immunomodulation of autoimmune and inflammatory diseases with intravenous immune globulin. N Engl J Med 2001;345:747–755. 11547745 10.1056/NEJMra993360

[2] Woodruff RK, Grigg AP, Firkin FC, Smith IL. Fatal thrombotic events during treatment of autoimmune thrombocytopenia with intravenous immunoglobulin in elderly patients. Lancet 1986;328:217–218. 10.1016/s0140-6736(86)92511-0 2873457

[3] Basta M. Intravenous immunoglobulin-related thromboembolic events: an accusation that proves the opposite. Clin Exp Immunol 2014;178:153–155. 25546802 10.1111/cei.12551 PMC4285531

[4] Rajabally YA, Kearney DA. Thromboembolic complications of intravenous immunoglobulin therapy in patients with neuropathy: a two-year study. J Neurol Sci 2011;308:124–127. 21679973 10.1016/j.jns.2011.05.035

[5] Ammann EM, Jones MP, Link BK, et al. Intravenous immune globulin and thromboembolic adverse events in patients with hematologic malignancy. Blood 2016;127:200–207. 26443622 10.1182/blood-2015-05-647552 PMC4713161

[6] NHS Digital. Hospital Episode Statistics, Admitted Patient Care: England, 2014–2015. 2015. Available at: digital.nhs.uk. Accessed December 1, 2016.

[7] NHS Digital. Hospital Episode Statistics, Admitted Patient Care: England, 2015–2016. 2016. Available at: digital.nhs.uk. Accessed January 23, 2017.

[8] Finnikin S, Ryan R, Marshall T. Statin initiations and QRISK2 scoring in UK general practice: a THIN database study. Br J Gen Pract 2017;67:e881–e887. 29061715 10.3399/bjgp17X693485 PMC5697558

